# Giant aneurysm of the atrial septum associated with premature closure of foramen ovale

**DOI:** 10.1186/1476-7120-3-20

**Published:** 2005-08-12

**Authors:** Constantinos Chrysostomou, Rita L Romaguera, Maria M Rodriguez

**Affiliations:** 1 Division of Cardiac Intensive Care, Department of Pediatrics, Children's Hospital of Pittsburgh, Pittsburgh, USA; 2 Department of Pathology, University of Miami / Jackson Memorial Hospital, Miami, USA

**Keywords:** Giant atrial septal aneurysm, premature closure of foramen ovale, heart failure, trisomy 18

## Abstract

Premature closure or restriction of foramen ovale (PCFO) is a rare congenital anomaly that can lead to a wide spectrum of cardiac malformations. This spectrum of secondary malformations appears to depend on the gestational timing of closure of the foramen ovale and to the degree of restriction. Earlier in the gestation, closure of the foramen has been associated with severe hypoplasia of the left ventricle whereas later closure has been associated with right heart failure and rarely with the formation of an aneurysm of the atrial septum. We describe the case of a 1 day old infant in whom PCFO resulted in severe right heart failure in addition to the formation of a giant atrial septal aneurysm.

## Case Report

**Figure 1:**
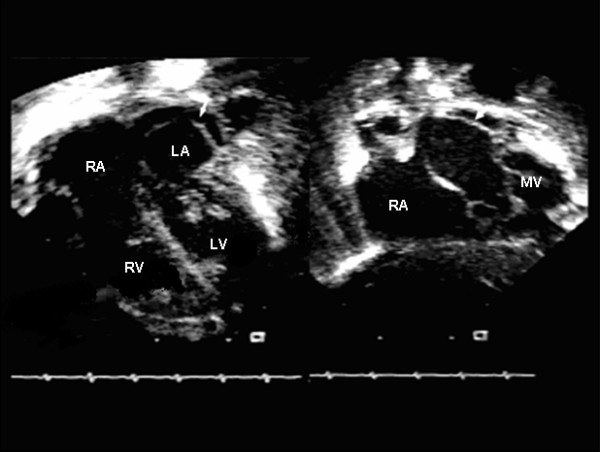
Apical four chamber and subcostal views. Note the membrane (arrow) extending from the level of the mitral valve to the upper pulmonary veins. RA; right atrium, LA; left atrium, RV; right ventricle, LV; left ventricle, MV; mitral valve.

A full term, small for gestational age infant (2.1 kg) without antenatal care was delivered urgently at our institution. Initial Apgar scores were 7 and 9 at 1 and 5 minutes respectively. Shortly, within less than 1 hour after delivery he developed significant respiratory distress (respiratory rate 70 breaths/min, retractions, nasal flaring) and symptoms and signs of right heart failure with tachycardia (heart rate 170 b/min) a prominent right ventricular impulse, hepatomegaly and ascites. Importantly this infant seemed to have the phenotypic features of Edwards's syndrome (trisomy 18) i.e. low birth weight, closed fists with index finger overlapping the 3rd digit, short sternum, rocker-bottom feet and microcephaly. Initial blood work up showed metabolic acidosis, with a pH: 7.23 and significant thrombocytopenia (platelet count: 58,000). An electrocardiogram was consistent with biventricular hypertrophy and the chest x-ray showed enlarged cardiac silhouette with increased pulmonary vascular markings. An echocardiogram was performed at 3 hours of life and demonstrated dysplastic and redundant atrioventricular and semilunar valves. There was moderate right atrial and right ventricular dilatation. The left atrium and left ventricle were mildly hypoplastic. The left ventricular ejection fraction was at low end of normal at 59% however there was a severely depressed right ventricular function. Of particular interest was the presence of an unusual, fixed, membrane-like structure occupying most of the left atrial cavity (Fig. 1). A small atrial septal wall gap was also present with bidirectional flow, as well as a small 3 mm, membranous ventricular septal defect (VSD). The ductus arteriosus was patent with bidirectional flow, indicating systemic pulmonary artery pressures. A small hemodynamically insignificant pericardial effusion was also noted. Inotropic support with dobutamine was initiated but several hours later the patient progressed into respiratory failure. After he was intubated unexpectedly he developed profound pulmonary hemorrhage. Despite multiple blood product transfusions and given the poor long term prognosis of trisomy 18, the patient was allowed to expire after parental consent.

**Figure 2:**
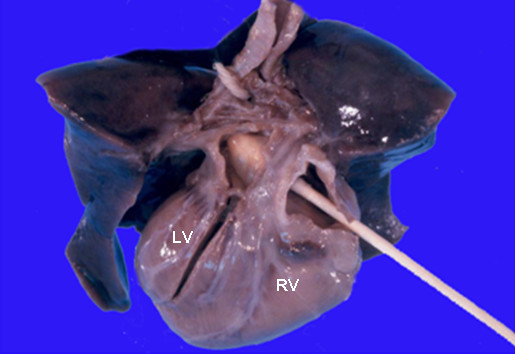
Heart and lungs block, posterior view. The right atrium is divided and the q-tip is directed from right to left through the fossa ovalis showing the giant atrial septal aneurysm (arrow) covering most of the left atrial cavity. The foramen ovale was closed. LV, left ventricle; RV, right ventricle.

The postmortem examination revealed the following: small pericardial effusion, bilateral moderate pleural effusions and ascites. The right sided cardiac structures were significantly dilated comparing with the left. The tricuspid valve annulus measured 37 mm comparing with the 10 mm mitral valve annulus and the pulmonary valve measured 24 mm comparing with the aortic valve of 8 mm. Both aortic and pulmonary valves were bicuspid. The left ventricle and left atrium (LA) were underdeveloped. The membrane seen within the LA on the echocardiogram was found to be a blind ended, wind sock like giant aneurysm of the atrial septum measuring 15 mm in length and 6 mm in width, occupying about 70% of the LA cavity (Fig. 2). What was thought to be a gap in the atrial septal wall with bidirectional shunt was a swirling of blood in and out of this blind ended aneurysm from the right atrial side through the fossa ovalis. There was a small membranous VSD and a moderate sized patent ductus arteriosus.

## Discussion

The importance of the foramen ovale (FO) during fetal life is well established, but the exact mechanism that causes it to close prematurely has been of some debate over the years. During normal fetal circulation 65% of the total caval blood flows through a FO to the left side, providing oxygenated blood both to the cerebral and coronary circulations [1]. However, in cases were the FO prematurely closes or becomes restrictive, a sequence of events may follow that can result in a spectrum of cardiac anomalies, the severity of which is believed to depend both on the gestational time the restriction occurs as well as on the extent of the restriction. This excess amount of blood flow that is re-directed to the right ventricle can result in congestive right heart failure and at the same time, because of the reduced blood flow to the left, the left sided structures can become hypoplastic [2,3]. Chronologically earlier closure of the FO seems to be related with a more profound underdevelopment e.g. hypoplastic left heart syndrome, whereas closure later in gestation can have only a minimal impact on the left heart. However, another theory that has been proposed as an explanation of PCFO, is that of abnormal hemodynamic development, such as with preexisting aortic or mitral valve stenosis. In these situations the elevated left atrial pressures may prematurely initiate the normal closure mechanism of the FO that pushes the flexible septum primum against the more rigid septum secundum [4-7].

In certain patients with PCFO, like in our case, there is a formation of an aneurysm of the atrial septum. In favor of the theory of primary PCFO, this aneurysm consistently bulges into the left atrium [8-10] whereas in the presence of left sided obstructive pathology, the aneurysm may bulge into the right atrium [4,11]. Furthermore, the presence of a giant septal aneurysm indicates a somewhat later closure of the foramen, explaining why in these situations the left side chambers are only mildly diminished in size. In addition, coexisting lesions like a VSD can provide a variable amount of flow to the left ventricle in terms of growth.

Though in this scenario the patient did not have the benefit or prenatal screening, PCFO can be diagnosed echocardiographically in-utero. The FO is defined as restrictive if its maximal diameter is less than 3 mm [5], however in the presence of a atrial septal aneurysm this can be somewhat more difficult to assess since the fossa ovalis is patent but the FO maybe closed or restrictive as in our case. Furthermore, an atrial septal aneurysm can be confused with other congenital anomalies e.g. cor triatriatum, in which management and prognosis are quite different.

A close monitoring for signs and symptoms of decompensation is recommended for both fetuses and infants with the diagnosis of PCFO. Prenatally, and in the phase of worsening heart failure an intervention may be warranted. Balloon atrial septostomy could be performed to improve the failing right ventricle by creating a pop-off communication at the atrial level, or to improve the elevated left atrial and pulmonary venous pressures, as in the case of hypoplastic left sided structures [12]. Also, like in other causes of in utero heart failure maternal digitalization may offer a significant improvement without the need of more radical intervention. Nonetheless, if above measures fail or not available then preterm delivery may be necessary. Postnatally, the course is determined mostly by the degree of prenatal compromise. While heart failure is expected to improve, some patients may require a significant amount of inotropic support.

## Authors' contributions

CC: Conceived of the study, participated in the study design and drafted the manuscript. RR: Carried out the pathology specimen investigation and participated in the study design.

MR: Carried out the pathology specimen investigation, participated in the study design and helped to draft the manuscript
